# Prediction of Plant Resistance Proteins Based on Pairwise Energy Content and Stacking Framework

**DOI:** 10.3389/fpls.2022.912599

**Published:** 2022-05-31

**Authors:** Yifan Chen, Zejun Li, Zhiyong Li

**Affiliations:** ^1^College of Computer Science and Electronic Engineering, Hunan University, Changsha, China; ^2^School of Computer Science and Technology, Hunan Institute of Technology, Hengyang, China

**Keywords:** plant resistance protein, pairwise energy content, discrete wavelet transform, stacking, feature representation

## Abstract

Plant resistance proteins (R proteins) recognize effector proteins secreted by pathogenic microorganisms and trigger an immune response against pathogenic microbial infestation. Accurate identification of plant R proteins is an important research topic in plant pathology. Plant R protein prediction has achieved many research results. Recently, some machine learning-based methods have emerged to identify plant R proteins. Still, most of them only rely on protein sequence features, which ignore inter-amino acid features, thus limiting the further improvement of plant R protein prediction performance. In this manuscript, we propose a method called StackRPred to predict plant R proteins. Specifically, the StackRPred first obtains plant R protein feature information from the pairwise energy content of residues; then, the obtained feature information is fed into the stacking framework for training to construct a prediction model for plant R proteins. The results of both the five-fold cross-validation and independent test validation show that our proposed method outperforms other state-of-the-art methods, indicating that StackRPred is an effective tool for predicting plant R proteins. It is expected to bring some favorable contribution to the study of plant R proteins.

## Introduction

The rapid multiplication and spread of pathogens affect plant growth and development and pose a serious threat to crop and food security. Resistance (R) proteins are of increasing interest because of their important role in plant defense against pathogens. R-proteins are plant proteins that contain a variety of structural domains such as nucleotide-binding structural domains (NB-ARC), leucine-rich repeat (LRR), Toll-interleukin-like receptor (TIR), Coiled-Coiled structures (CC), and kinases (KIN) (Sanseverino and Ercolano, 2012; Kushwaha et al., 2016). The exploration of R-proteins and proteins with R-protein characteristics can play a key role in plant defense against different pathogens. In recent years, computational methods have been widely used in R-protein prediction studies.

Currently, computational methods for predicting R-proteins fall into two main categories: sequence alignment-based and machine-learning-based methods. The main methods based on sequence alignment are NLR-parser (Steuernagel et al., 2015), RGAugury (Li et al., 2016), and Restrepo-Montoya’s pipeline (Restrepo-Montoya et al., 2020).

NLR-parser predicts NLR-like sequences based on MAST motif search (Steuernagel et al., 2015). RGAugury predicts different R protein subclasses by integrating the results generated by several computational tools (Li et al., 2016), including the following: BLAST (Camacho et al., 2009), Hmmer3 (Eddy, 2011), Phobius (Käll et al., 2004), TMHMM (Bateman et al., 2004), and so on. Restrepo-Montoya et al. (2020) developed a computational approach to classify RLK and RLP proteins using SignalP 4.0 (Petersen et al., 2011), TMHMM 2 (Krogh et al., 2001), and PfamScan (Finn et al., 2014). In general, sequence alignment-based methods generally have low sensitivity and are time-consuming, which makes them difficult to predict proteins with low similarity. The application of machine learning methods for predicting plant R proteins has thus become of increasing interest.

Machine learning methods have been widely used to study plant and animal protein data (Sun et al., 2020a,b, 2021). Several common machine learning-based methods for predicting R proteins are described below: NBSPred (Kushwaha et al., 2016), DRPPP (Pal et al., 2016), prPred (Wang et al., 2021b), and prPred-DRLF (Wang et al., 2022). The NBSPred (Kushwaha et al., 2016) method is a high-throughput pipeline based on support vector machine (SVM), which is used to identify NBSLRR and NBSLRR-like proteins from non-NBSLRR proteins from genomic, transcriptomic and protein sequences, and was tested and validated employing input sequences from three dicots and two monocot plants. Similarly, the DRPPP (Pal et al., 2016) method is an SVM-based predictive approach to predict disease resistance proteins in plants. The method applied 16 feature methods to obtain 10,270 features and performed ten-fold cross-validation to train optimized radial basis function SVM parameters, achieving an overall accuracy of 91.11% on the test dataset. Recently, two machine learning-based methods, prPred (Wang et al., 2021b) and prPred-DRLF (Wang et al., 2022), were proposed by Wang et al. to predict Plant R proteins. prPred (Wang et al., 2021b) used two feature extraction methods, k-spaced amino acid pairs (CKSAAPs) and k-spaced amino acid group pairs (CKSAAGPs), to obtain Plant R protein sequence feature information, and then used a two-step feature selection strategy to detect irrelevant and redundant features. The prediction accuracy of the prPred model was 93.5%. The prPred-DRLF method applied bi-directional long short-term memory (BiLSTM) and unified representation (UniRep) embedding to represent Plant R protein sequence features and used a light gradient boosting machine (LGBM) classifier to identify plant R proteins, achieving a prediction accuracy of 95.6% in independent tests.

Although considerable progress has been made in existing machine learning methods for predicting Plant R proteins, some significant challenges remain. For example, most prediction methods only target the sequence features of Plant R proteins, ignoring the protein structure and the physicochemical properties of the bases. In contrast, protein residue pairwise energy content matrices (RECM) have been used to predict intrinsically non-structural proteins due to their ability to capture energy information between residue pairs (Jones and Cozzetto, 2015; Mészáros et al., 2018). Mishra et al. (2019) used the characteristics of protein residue pair energy content to predict DNA and RNA binding proteins, and Fu et al. (2020) used the characteristics of protein residue pair energy content to predict cell-penetrating peptides.

In recent years, the Stacking framework has been widely used in biological sequence prediction, including protein, non-coding RNA and RNA-protein interaction prediction, etc. Mishra et al. (2019) proposed a method for predicting DNA-binding proteins by combining evolutionary information and a stacking framework; Yi et al. (2020) proposed a method to predict ncRNA-protein interactions by fusing multiple sources of information and the stacking framework; Fu et al. (2020) used the stacking framework to construct a prediction model for cell-penetrating peptides and their uptake efficiency; Basith et al. (2022) applied 11 different encodings to represent three different features and input them into the stacking model to predict prokaryotic lysine acetylation sites; Wang et al. (2021a) proposed a hybrid framework based on a stacking strategy to predict non-coding RNAs.

In this manuscript, we propose a machine learning-based predictor, called StackRPred, to further improve Plant R protein prediction accuracy. The main contributions of StackRPred are as follows.

(i) We employ RECM to encode Plant R proteins and combine the discrete wavelet transform (DWT) (Shensa, 1992) and pseudo position-specific score matrix (PsePSSM) (Chou and Shen, 2007) to obtain Plant R protein feature representations. (ii) We used a stacking-based machine learning model to efficiently predict Plant R proteins. The model consists of two layers; the first layer (base layer) uses these features to train an ensemble of predictors; the second layer (meta-layer) combines the outputs of the predictors from the base layer. (iii) The prediction results show that StackRPred outperforms state-of-the-art methods for Plant R protein prediction. The superior performance of StackRPred could motivate researchers to explore Plant R proteins even further.

## Datasets and Methods

### Framework of the Proposed Method

**FIGURE 1:**
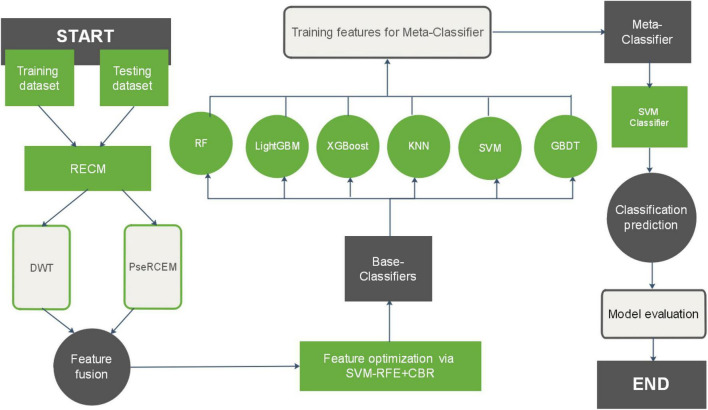
Overview of the StackRPred procedure.

In this study, we present a sequence-based plant R protein prediction model called StackRPred. The StackRPred prediction model consists of two major parts, feature extraction and classifier construction. (1) Feature extraction; we first calculate the RECM matrix (see Section “Residue Pairwise Energy Content Matrices”) of Plant R protein in the benchmark dataset according to the physicochemical properties of the Plant R protein sequence, and extract the PsePSSM and DWT characteristics of each Plant R protein based on the RECM matrix. Then, we use SVM-RFE + CBR (Yan and Zhang, 2015) method to reduce the dimensionality of the feature information. (2) Classifier construction; We constructed a stacking model to build the classification model. Our proposed Stacking model classifier consists of two layers: the first layer (base layer) contains multiple classifiers; the second layer includes one classifier called the meta-layer. The base layer consists of eXtreme Gradient Boosting (XGBoost), SVM, K-Nearest Neighbor (KNN), Gradient Boosting Decision Tree (GBDT), Light Gradient Boosting Machine (LightGBM), and Random Forest (RF); the meta-layer uses SVM as the meta-classifier. The overall framework of the proposed method for predicting Plant R protein is shown in Figure 1.

### Datasets

The dataset used in this thesis was derived from the study by Wang et al. (2021b). The specific data were obtained as follows: R proteins of 35 plant species were obtained from the PRGdb database (Osuna-Cruz et al., 2018) and the protein sequences of these 35 plant species were downloaded from the NCBI database to construct a positive sample dataset; then, proteins with sequence similarity greater than 30% were excluded from the non-R protein dataset using CD-HIT (Fu et al., 2012). The obtained dataset contains 456 protein sequences with 152 positive and 304 negative samples. The data set is divided into a training sample set and a test sample set with a ratio of 8:2, the number of training samples is 364, and the number of independent test samples is 92. The training dataset consisted of 121 plant R protein sequences and 243 non-R protein sequences; the independent test dataset consisted of 31 R protein sequences and 61 non-R protein sequences.

### Residue Pairwise Energy Content Matrices

The energy of interaction between protein residues ensures protein structural stability, and the energy contribution of residue interactions can be approximated by an energy function extracted from known structures (Hoque et al., 2016; Mishra et al., 2016). Dosztanyi et al. (2005) performed the least square fit of the contact energy derived from the primary sequences of 674 proteins to the tertiary structures of 785 proteins and constructed the RCEM matrix, a 20×20 dimensional matrix with rows and columns representing the 20 standard amino acids. Table 1 shows the RCEM table applied in this manuscript (Dosztanyi et al., 2005).

**TABLE 1:** The residue pairwise energy content matrices (RECM).

	A	C	D	E	F	G	H	I	K	L	M	N	P	Q	R	S	T	V	W	Y
A	–1.65	–2.83	1.16	1.8	–3.73	–0.41	1.9	–3.69	0.49	–3.01	–2.08	0.66	1.54	1.2	0.98	–0.08	0.46	–2.31	0.32	–4.62
C	–2.83	–39.58	–0.82	–0.53	–3.07	–2.96	–4.98	0.34	–1.38	–2.15	1.43	–4.18	–2.13	–2.91	–0.41	–2.33	–1.84	–0.16	4.26	–4.46
D	1.16	–0.82	0.84	1.97	–0.92	0.88	–1.07	0.68	–1.93	0.23	0.61	0.32	3.31	2.67	–2.02	0.91	–0.65	0.94	–0.71	0.90
E	1.8	–0.53	1.97	1.45	0.94	1.31	0.61	1.3	–2.51	1.14	2.53	0.2	1.44	0.1	–3.13	0.81	1.54	0.12	–1.07	1.29
F	–3.73	–3.07	–0.92	0.94	–11.25	0.35	–3.57	–5.88	–0.82	–8.59	–5.34	0.73	0.32	0.77	–0.4	–2.22	0.11	–7.05	–7.09	–8.80
G	–0.41	–2.96	0.88	1.31	0.35	–0.2	1.09	–0.65	–0.16	–0.55	–0.52	–0.32	2.25	1.11	0.84	0.71	0.59	–0.38	1.69	–1.90
H	1.9	–4.98	–1.07	0.61	–3.57	1.09	1.97	–0.71	2.89	–0.86	–0.75	1.84	0.35	2.64	2.05	0.82	–0.01	0.27	–7.58	–3.20
I	–3.69	0.34	0.68	1.3	–5.88	–0.65	–0.71	–6.74	–0.01	–9.01	–3.62	–0.07	0.12	–0.18	0.19	–0.15	0.63	–6.54	–3.78	–5.26
K	0.49	–1.38	–1.93	–2.51	–0.82	–0.16	2.89	–0.01	1.24	0.49	1.61	1.12	0.51	0.43	2.34	0.19	–1.11	0.19	0.02	–1.19
L	–3.01	–2.15	0.23	1.14	–8.59	–0.55	–0.86	–9.01	0.49	–6.37	–2.88	0.97	1.81	–0.58	–0.6	–0.41	0.72	–5.43	–8.31	–4.90
M	–2.08	1.43	0.61	2.53	–5.34	–0.52	–0.75	–3.62	1.61	–2.88	–6.49	0.21	0.75	1.9	2.09	1.39	0.63	–2.59	–6.88	–9.73
N	0.66	–4.18	0.32	0.2	0.73	–0.32	1.84	–0.07	1.12	0.97	0.21	0.61	1.15	1.28	1.08	0.29	0.46	0.93	–0.74	0.93
P	1.54	–2.13	3.31	1.44	0.32	2.25	0.35	0.12	0.51	1.81	0.75	1.15	–0.42	2.97	1.06	1.12	1.65	0.38	–2.06	–2.09
Q	1.2	–2.91	2.67	0.1	0.77	1.11	2.64	–0.18	0.43	–0.58	1.9	1.28	2.97	–1.54	0.91	0.85	–0.07	–1.91	–0.76	0.01
R	0.98	–0.41	–2.02	–3.13	–0.4	0.84	2.05	0.19	2.34	–0.6	2.09	1.08	1.06	0.91	0.21	0.95	0.98	0.08	–5.89	0.36
S	–0.08	–2.33	0.91	0.81	–2.22	0.71	0.82	–0.15	0.19	–0.41	1.39	0.29	1.12	0.85	0.95	–0.48	–0.06	0.13	–3.03	–0.82
T	0.46	–1.84	–0.65	1.54	0.11	0.59	–0.01	0.63	–1.11	0.72	0.63	0.46	1.65	–0.07	0.98	–0.06	–0.96	1.14	–0.65	–0.37
V	–2.31	–0.16	0.94	0.12	–7.05	–0.38	0.27	–6.54	0.19	–5.43	–2.59	0.93	0.38	–1.91	0.08	0.13	1.14	–4.82	–2.13	–3.59
W	0.32	4.26	–0.71	–1.07	–7.09	1.69	–7.58	–3.78	0.02	–8.31	–6.88	–0.74	–2.06	–0.76	–5.89	–3.03	–0.65	–2.13	–1.73	–12.39
Y	–4.62	–4.46	0.9	1.29	–8.8	–1.9	–3.2	–5.26	–1.19	–4.9	–9.73	0.93	–2.09	0.01	0.36	–0.82	–0.37	–3.59	–12.39	–2.68

### Discrete Wavelet Transform Features

Discrete Wavelet Transform (DWT) (Shensa, 1992) is a transform operation that can capture wavelet discrete sampling of sequence base frequency and position information. The transform operation is done by projecting the signal onto the wavelet function. When applied to Plant R protein sequence analysis, DWT can decompose the physicochemical properties of the base sequence into a list of coefficients of different resolutions and also remove noise information from the high-pass curve. In this manuscript, the RECM matrix is calculated for each given Plant R protein sequence. Then, each RECM matrix is regarded as a two-dimensional signal, and the whole of the two-dimensional signal is denoised by discrete wavelet transform.

Wavelet transform (WT) is defined as the projection of the signal f(t) onto the wavelet function:


(1) $$T\,\left(a,b\right)=\frac{1}{\sqrt{a}}\,\int_{a}^{t}f\,\left(t\right)\,\Psi \,\left(\frac{t-b}{a}\right)\,\mathrm{dt}$$

Where a(a > 0) is a scale factor and b is a translation factor, and both belong to the real set r(n). $\Psi \,\left(\frac{t-b}{a}\right)$ is the analyzing wavelet function, and T(a, b) is the wavelet transform coefficient of the signal at the specific position (t = b) and the specific wavelet period (the equation of the scale factor a). Discrete wavelet transform (DWT) can decompose lncRNA sequences into coefficients of different dilations and then remove noise components. Nanni et al. (2012, 2014) proposed an efficient algorithm for performing DWT by assuming that the discrete signal f(t) is x [n], and is defined as follows:


(2) $$y_{j,l\,o\,w}\,\left[n\right]=\sum_{k=1}^{N}x\,\left[k\right]\,g\,\left[2\,n-k\right]$$

(3) $$y_{j,h\,i\,g\,h}\,\left[n\right]=\sum_{k=1}^{N}x\,\left[k\right]\,h\,\left[2\,n-k\right]$$

Where N is the length of the discrete signal. *y*_*low*_[*n*] is the approximation coefficient of the signal (low frequency component). *y*_*high*_[*n*] is the detailed coefficient (high frequency component). g is a low pass filter and h is a high pass filter. As the level of decomposition increases, more detailed signal characteristics can be observed.

**FIGURE 2:**
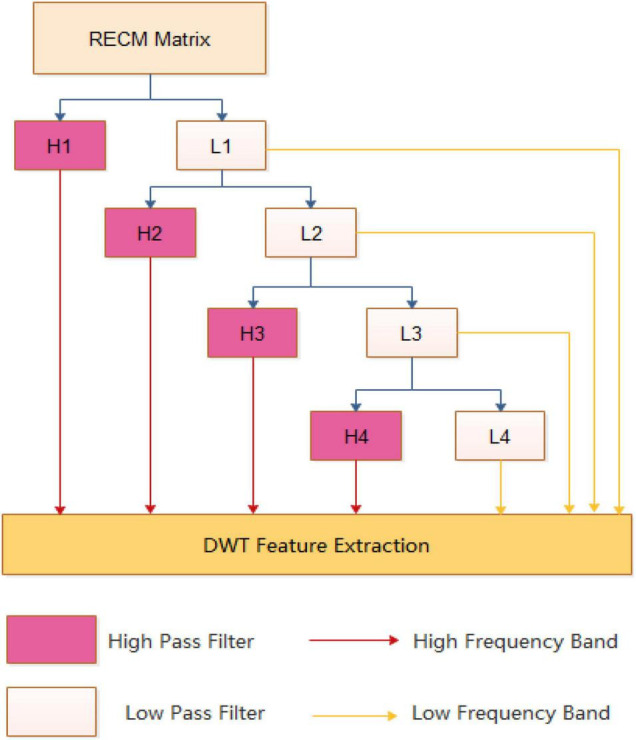
An example of a discrete wavelet transform process.

Figure 2 is an example of a 4-level discrete wavelet transform. At each level, the data can be divided into a high frequency band containing more noise information and a low frequency band including more useful signals, and should be transformed in the next stage.

At each level of the DWT, the high and low band signals are separated. Inspired by the work of Nanni et al. (2012, 2014), we calculate the maximum, minimum, mean, and standard deviation values for each band. Four characteristics can be obtained for the high frequency and the low frequency, respectively, and a total of eight features are obtained. In addition, since the high-frequency component noise is large, the low-frequency component is more important. We also extract the first five discrete cosine coefficients from the approximation coefficients, and the first five elements are more important information indicating the sequence in the compressed low-band. Therefore, we can get 4 + 4 + 5 features from each level of DWT, and there are 52 features of 5 levels throughout the conversion process.

In the RECM matrix, we can extract 52 features for each attribute using the 5-level DWT method. Thus, we can obtain 1040 features.

### PsePSSM Features

Chou and Shen (2007b) proposed the Pseudo Position-Specific Score Matrix (PsePSSM) feature extraction method widely used for protein sequence feature extraction. Similarly, we established a new feature extraction method based on RECM matrix–PseRECM, which can be used for feature extraction of Plant R protein sequences. PseRECM is defined as follows.


(4) $$P\,R_{P\,s\,e\,R\,C\,E\,M}^{\lambda }=\left(\bar{P_{1}^{\prime }},\bar{P_{2}^{\prime }},\ldots ,\bar{P_{20}^{\prime }},G_{1}^{1},G_{2}^{1},\ldots ,G_{20}^{1},\ldots ,G_{1}^{\lambda },G_{2}^{\lambda },\ldots ,G_{20}^{\lambda }\right)$$

Where


(5) $$\bar{P_{j}}=\frac{\sum_{i=1}^{L}p_{i,j}}{L},1\leq i\leq L,j=1,2,\ldots \,20$$

Here *p*_*i*,*j*_ represents the values of the *i*-th row and the *j*-th column in the RECM matrix.


(6) $$G_{j}^{\lambda }=\frac{\sum_{i=1}^{L-\lambda }{\left(p_{i,j}-p_{i+\lambda ,j}\right)}^{*}\,\left(p_{i,j}-p_{i+\lambda ,j}\right)}{L-\lambda }$$

Where $G_{j}^{\lambda }$ is the average correlation of amino acid residues with a separation distance λ (λ < L) in the sequence, *j* = 1, 2,…, 20.

### Feature Optimization Algorithm

After extracting feature information for the full Plant R protein dataset, to eliminate noise and redundant features from the original feature space and reduce overfitting to improve performance, we employ the SVM-RFE + CBR (Yan and Zhang, 2015) algorithm to select the best feature subset. the SVM-RFE + CBR (Yan and Zhang, 2015) algorithm has been successfully applied to many systems biology problems (Fu et al., 2018, 2019a,b; Chen et al., 2021). We first use SVM-RFE + CBR to rank all feature vectors and select a set of top-ranked feature vectors, and then, reorganize the selected feature vectors into new and ordered feature vectors. The 112-dimensional feature input model is obtained for training after applying the SVM-RFE + CBR algorithm.

The SVM-RFE algorithm is an Embedded method based on the maximum interval principle of SVM, proposed by Guyon et al. in the classification of cancer, and has been successfully applied to many systems biology problems (Yin et al., 2016; Chowdhury et al., 2017). The SVM-RFE algorithm trains samples through the model and ranks the score of each feature, removes the feature with the lowest score, then trains the model again with the remaining features for the next iteration, and finally selects the number of features needed. To reduce the potential bias between non-linearity and linearity of the SVM-RFE algorithm, Yan et al. incorporated the Correlation Bias Reduction (CBR) strategy and proposed the SVM-RFE + CBR algorithm. To incorporate the CBR strategy into the feature elimination process, half of the remaining features are removed in each iteration of SVM-RFE at the beginning of the algorithm. When the number of remaining features is less than an elimination threshold, they are removed in the next iterations for better accuracy.

The SVM-RFE + CBR algorithm requires the following main parameters: kerType, rfeC, rfeG, useCBR, Rth. The values and descriptions of these parameters in this paper are shown in Table 2.

**TABLE 2:** Parameters description in SVM-RFE + CBR method.

Parameter	Value	Describe
kerType	2	Kernel type, see libsvm. linear: 0; rbf:2
rfeC	16	Parameter C in SVM training
rfeG	0.0078	Parameter g in SVM training
useCBR	True	Whether or not use CBR
Rth	0.9	Corrcoef threshold for highly corr features

### Classification Models

The stacking method achieves model stacking by combining the output results of multiple models (called base models) as feature input to the next layer of models. Specifically, the model output of the first layer of the stacking model is used as the input of the second layer of the model, the output of the second layer of the model is used as the input of the third layer of the model, and so on, with the output of the last layer of the model as the final result. In this manuscript, a stacking model is constructed as a classification prediction model for Plant R proteins. The StackRPred model consists of two layers: the first layer (base layer) contains multiple classifiers. The classifier in the base layer is called the base classifier; the second layer includes one classifier called the meta layer. The output of the base classifier is used as input data for the meta-classifier in the overlay model, so that the meta-classifier can be found and corrected for deviations in the base classifier and learn inductively from the results of the base classifier, thus improving the generalization accuracy of the integrated classifier. Choosing suitable base classifiers and meta-classifiers is the key to improving the generalization ability of the StackRPred model. In this study, through several experimental tests, we selected six classification algorithms as the base classifier for the first layer, namely KNN, GBDT, SVM, XGBoost, LightGBM, and RF, and chose SVM as the meta-classifier.

K-nearest neighbor (Altman, 1992) is a non-parametric statistical method for classification and regression. The core idea of KNN: If most of the K nearest neighbors of a sample in the feature space belong to a certain category, the sample also belongs to this category and has the characteristics of the samples in this category. This method only determines the category of the sample is classified according to the category of the nearest one or several samples in determining the classification decision.

Support vector machine (Vapnik, 1999) is a generalized linear classifier that classifies data by supervised learning, and its decision boundary is the maximum-margin hyperplane for solving learning samples. In this study, we employ grid search to optimize the RBF kernel parameter γ and the cost parameter C, and choose the radial basis function (RBF) as the SVM kernel function.

Gradient boosting decision tree (Friedman, 2001) is an iterative decision tree algorithm that consists of multiple decision trees, with the conclusions of all the trees accumulating to make the final decision. It was considered to be a more generalizable algorithm when it was first proposed, along with SVM.

Random forest (Svetnik et al., 2003) is a classifier that contains multiple decision trees and whose output classes are determined by the plurality of the classes output by the individual trees. RF randomly combines multiple decision trees into a forest, and determines the final class of the test sample based on the voting results of each decision tree during classification.

eXtreme gradient boosting (Chen and Guestrin, 2016) is an algorithm that integrates and boosts multiple weak classifiers into a strong classifier. Compared to gradient boosting classifier (GBC), XGBoost performs more regularized model formalism to control model overfitting, thus improving performance.

LightGBM is a gradient-lifting tree framework proposed by Ke et al. (2017). LightGBM is a framework for implementing the GBDT algorithm, which supports efficient parallel training and has the advantages of faster training speed, lower memory consumption, better accuracy, and distributed support for fast processing large amounts of data.

The following describes the settings of the parameters in the six classifiers.

XGBoost/RF: The number of trees in the model is fine-tuned using the grid search method, i.e., the value of the “n_estimators” variable and the rest of the parameters are default parameters.


100≤n_estimators≤1000⁢w⁢i⁢t⁢h⁢s⁢t⁢e⁢p⁢△⁢n⁢_⁢estimators=25

SVM: We choose the radial basis function as the kernel function of the SVM and use the grid search to optimize the parameters *C* and γ. Therefore, we optimized these parameters using the following range:


$$\left\{ \begin{array}{c} 2^{-5}\leq C\leq 2^{15}\,w\,i\,t\,h\,s\,t\,e\,p\,△\,C=2\\ 2^{-15}\leq \gamma \leq 2^{15}\,w\,i\,t\,h\,s\,t\,e\,p\,△\,\gamma =2^{-1} \end{array}\right.$$

LightGBM: Fine-tune the three key parameters “n_estimators,” “max_depth,” and “learning_rate” in the model using the grid search method:


$$\left\{ \begin{array}{c} 100\leq \text{n\_estimators}\leq 1000\,w\,i\,t\,h\,s\,t\,e\,p\,\text{n\_estimators}=25\\ 1\leq \text{max\_depth}\leq 25\,w\,i\,t\,h\,s\,t\,e\,p\,△\,\text{max\_depth}=1\\ 0.1\leq \text{learning\_rate}\leq 0.8\,w\,i\,t\,h\,s\,t\,e\,p\,△\,\text{learning\_rate}=0.01 \end{array}\right.$$

KNN/GBDT: All parameters are default values.

## Experiments and Results

### Evaluation Criteria

To evaluate the performance of the proposed plant R protein prediction model, four metrics were introduced in this study to evaluate the performance of the model prediction. These four evaluation metrics are: Precision, Recall, Accuracy (ACC), and F1-score, which are formulated as follows.


(7) $$\text{Recall}=\frac{T\,P}{T\,P+F\,N}$$

(8) $$\text{Precision}=\frac{T\,P}{T\,P+F\,P}$$

(9) $$F_{1}-s\,c\,o\,r\,e=2\times \frac{R\,e\,c\,a\,l\,l\times \mathrm{Pr}e\,c\,i\,s\,i\,o\,n}{R\,e\,c\,a\,l\,l+\mathrm{Pr}e\,c\,i\,s\,i\,o\,n}$$

(10) $$A\,C\,C=\frac{T\,P+T\,N}{T\,P+F\,P+T\,N+F\,N}$$

Where TP, TN, FP, and FN denote the number of true positives, true negatives, false positives, and false negatives, respectively.

In addition, receiver operating characteristics (ROC) were plotted on the basis of specificity and sensitivity, and the area under the ROC curve (AUC) was calculated on the basis of the trapezoidal approximation. The AUC provides a measure of classifier performance; large values of AUC correspond to improved classifier performance.

### Measuring Algorithm Performance Through Five-Fold Cross-Validation

K-fold cross-validation is one of the most common ways to measure the performance of a computational model. In this manuscript, we apply five-fold cross-validation to the training set and calculate the evaluation metrics of Accuracy, Sensitivity, Precision, Specificity and AUC. The average experimental results for the five-fold cross-validation of these evaluation metrics are as follows: Accuracy (0.975), Precision (0.984), Recall (0.942), and AUC (0.995). prPred-DRLF model uses the three optimizations of LGBM, RF, and MRMD3.0 (Zou et al., 2016; He et al., 2020). The best accuracies corresponding to these three optimization algorithms for the prPred-DRLF model are 0.97, 0.964, and 0.964, respectively, all of which are lower than the accuracy achieved by our method (0.975).

**FIGURE 3:**
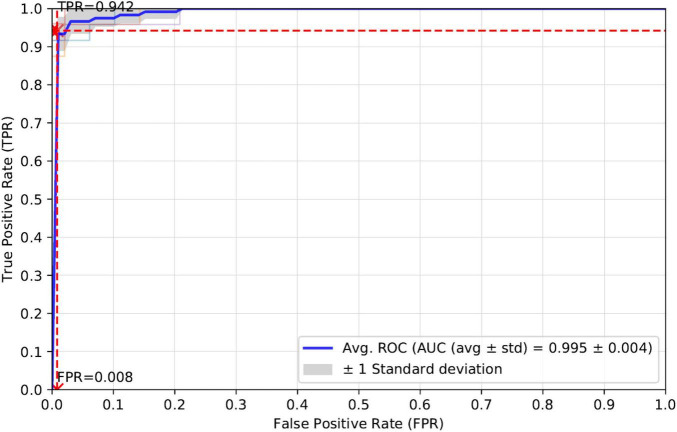
ROC curves for five-fold cross-validation of our proposed model.

Therefore, a comparison of the experimental results shows that our proposed model is superior to the prPred-DRLF model. It is worth mentioning that the prPred-DRLF model extracts far more feature dimensions than our method, indicating that our method uses fewer feature dimensions and is able to capture more effective feature information. For a better presentation of the results, we plotted the average ROC curve for the five-fold cross-validation, as shown in Figure 3.

### Measuring Algorithm Performance Through Independent Test Validation

To further compare the performance of our proposed method with other methods in independent tests, we compared it with the prPred and prPred-DRLF groups of methods, respectively, and chose the best experimental results given in their papers for these two models. The experiments were compared under the same dataset and the results are shown in Table 3. The prPred model has an accuracy value of 0.935 and a Precision value of 1 which is the largest among all the models. A total of three features were extracted from the prPred-DRLF model, namely TAPE-BERT, BiLSTM, and UniRep. prPred-DRLF1 in Table 3 represents the result of the prPred-DRLF model choosing the combination of BiLSTM + UniRep, which is the best accuracy given by the prPred-DRLF model. prPred-DRLF2 indicates the result of the prPred-DRLF model choosing all three combinations (TAPE-BERT, BiLSTM, and UniRep), which in contrast does not perform as well as prPred-DRLF1.

**TABLE 3:** Performance comparison with other state-of-the-art prediction methods on independent datasets.

Models	Accuracy	Precision	Recall	F1-score	AUC
prPred	0.935	1.000	0.806	0.893	0.948
prPred-DRLF1	0.956	0.967	0.905	0.933	0.997
prPred-DRLF2	0.923	0.943	0.838	0.884	0.989
StackRPred	0.967	0.980	0.968	0.980	0.997

**FIGURE 4:**
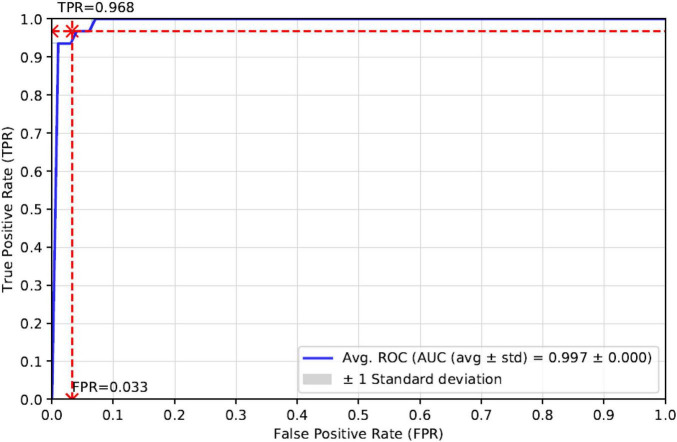
ROC curves for independent test validation of our proposed model.

As can be seen in Table 3, the Accuracy, Precision, Recall, F1-score, and AUC of our proposed method StackRPred were 0.967, 0.980, 0.968, 0.980, and 0.997, respectively, of which, except for Precision, all were maximum values, indicating the superiority of our method in predicting plant R proteins. Also, to make the results of our method more visual, we plotted the ROC curves, as shown in Figure 4.

## Conclusion

The discovery and study of plant R proteins is of great importance to agricultural production. In this study, we propose a novel plant R-protein predictor, StackRPred, which introduces DWT and PsePSSM methods to extract plant R-protein feature information based on the base pair energy content, and then applies SVM-RFE + CBR techniques to optimally select the obtained feature information to obtain 112-dimensional feature information; finally, the 112-dimensional feature information was fed into the constructed stacking model for training to build the prediction model. The stacking model was divided into two layers, with the first layer containing six classifiers, namely KNN, GBDT, SVM, XGBoost, LightGBM and RF, and the SVM was selected as the classifier in the second layer. Precision, Recall, Accuracy (ACC), F1-score, and AUC were used to evaluate the performance of the model, and a five-fold cross-validation and independent test validation were performed, respectively. The experimental results show that the proposed StackRPred model outperforms other state-of-the-art algorithms. The StackRPred model is useful for further exploration of plant R proteins and is expected to be extended to other protein or peptide research areas. In the future, we will focus more on the interpretability of plant R protein prediction models. Model interpretability is one of the key directions of current bioinformatics research (Cai et al., 2021a,b). The exploration of model interpretability is beneficial to further functional studies on plant R proteins.

## Data Availability Statement

The original contributions presented in the study are included in the article/supplementary material, further inquiries can be directed to the corresponding author.

## Author Contributions

YC and ZhL performed the experiments. YC wrote the manuscript. All authors conceived the concept of the work, contributed to the article, and approved the submitted version.

## Conflict of Interest

The authors declare that the research was conducted in the absence of any commercial or financial relationships that could be construed as a potential conflict of interest.

## Publisher’s Note

All claims expressed in this article are solely those of the authors and do not necessarily represent those of their affiliated organizations, or those of the publisher, the editors and the reviewers. Any product that may be evaluated in this article, or claim that may be made by its manufacturer, is not guaranteed or endorsed by the publisher.
